# The value of cervical screening to young women

**DOI:** 10.1038/sj.bjc.6601596

**Published:** 2004-03-02

**Authors:** A Herbert

**Affiliations:** 1 Chairman, British Society for Clinical Cytology, Histopathology Department, Second Floor, North Wing, St Thomas’ Hospital, Lambeth Palace Road, London SE1 7EH, UK

**Sir**,

The recent article by Sasieni *et al* is to be welcomed in that it supports previous evidence that 3-yearly screening may be more effective than 5-yearly in the prevention of cervical cancer, at least in women under 50 years of age (Sasieni *et al*, 2003). However, the conclusions about the effectiveness of screening in different age groups are not entirely justified from the data collected. For instance, comparison of cervical smear histories in women with and without cancer becomes less useful as a measure of screening effectiveness when screening coverage is high in the population at risk, and when the number of cancers that are screen-detected is not known.

The data and conclusions may be questioned for the following reasons. The screening histories cover different periods of time, some going back to the late 1980s, and do not account for significant changes in practice and quality control that have taken place since the 1990s. The screening histories in the control group are not what would be expected from a normal population. In Table 6 (Sasieni *et al*, 2003), 18.4% had never had a smear, whereas in England only 7% were recorded as having ‘no record’ of a test even as early as 1994–95 (Department of Health, 2001). The percentage of cancer cases with no record of a test is not given for comparison, because Table 6 includes recent symptomatic and diagnostic tests taken within 6 months. Cases in which the stage of cancer was unknown have been excluded, although these would almost certainly have represented fully invasive rather than microinvasive cancers. Women whose latest negative smear was reported 3 or 5 years before diagnosis were assumed to have been screened 3- or 5-yearly, when no information was provided about their previous screening history. It is not only the interval since the latest test that counts, but also the number of rounds of screening a woman has experienced during the years in which CIN might have been detected. Extending the interval to 5-yearly in women over 50 is probably only justified in women who have been screened regularly before that age.

It is misleading to conclude that screening is less effective at preventing frankly invasive cancer in women under 40 when its mechanism lies in the treatment of high-grade cervical intraepithelial neoplasia (CIN). In 1998, 90% of cases of CIN3 were detected in women aged less than 45 and approximately 70% in women aged less than 35. More than 4000 cases of CIN3 (15% of all cases) were detected in women under 25 (Cancer Research UK, 2003). How many of those women would develop cancer if they were left untreated? In how many would the CIN3 become so extensive that it would be difficult to excise, which is often the case by the time microinvasive cancer develops?

If the aim of cervical screening is to detect *pre-cancer*, the relative risk suggesting that screening has little effect in preventing *cancer* in young women is irrelevant. Invasive cancer in young women is rare and nowadays most of the cases are screen-detected. These include microinvasive cancers, which have been excluded by Sasieni *et al*. In the 12-year study of cancers in Southampton between 1985 and 1996, referred to by Sasieni *et al*, there was a reversal of the ratio of symptomatic: screen-detected cancers in women aged 25–34 years from 10 : 4 in 1985–87 to 4 : 8 in 1994–96, but no fall in the numbers of cancers in that age group (Herbert *et al*, 2001). This is a clinically important benefit of screening young women because 90% of screen-detected cancers were diagnosed at stage I, giving an excellent prognosis with respect to life expectancy (Herbert *et al*, 2001).

I can see no evidence from this study to suggest that women aged 20–24 should not be screened, bearing in mind that not all women would be screened as soon as they were 25. Treatment of high-grade CIN in young women reduces the risk of symptomatic cancer presenting later in life, after an interval that is unpredictable for an individual woman.
